# Fragmented QRS on Baseline Electrocardiography Predicts Anatomically Significant Stenosis in Patients With Intermediate Coronary Lesions on Coronary Computed Tomography Angiography

**DOI:** 10.14740/cr2284

**Published:** 2026-08-31

**Authors:** Eyyup Tusun, Mehmet Han Mercan, Muslum Karakas, Necmettin Korucuk, Veysel Tosun

**Affiliations:** a Department of Cardiology, Mehmet Akif Inan Training and Research Hospital, Sanliurfa, Turkiye; b Department of Cardiology, Antalya City Hospital, Antalya, Turkiye

**Keywords:** Fragmented QRS, Coronary computed tomography angiography, Intermediate stenosis, Invasive coronary angiography, Electrocardiography, Coronary artery disease

## Abstract

**Background:**

Intermediate-grade stenosis (40–70%) on coronary computed tomography angiography (CCTA) often presents diagnostic uncertainty regarding the necessity of invasive coronary angiography (ICA). Although fragmented QRS (fQRS) on electrocardiography (ECG) indicates myocardial scarring and ischemia, its predictive value remains unclear in this specific cohort. We investigated the association between baseline fQRS and anatomically severe stenosis (≥ 70%) confirmed by ICA.

**Methods:**

This study retrospectively evaluated 200 patients with CCTA-detected intermediate stenosis who underwent subsequent ICA. Patients were stratified based on angiographic findings into severe stenosis (≥ 70%, n = 89) and non-severe stenosis (< 70%, n = 111) groups. Standard 12-lead ECGs were analyzed, and independent predictors were determined using binary logistic regression and receiver operating characteristic (ROC) curves.

**Results:**

The prevalence of fQRS was significantly higher in the severe stenosis group (65.2% vs. 16.2%, P < 0.001). Diabetes mellitus (36.0% vs. 22.5%, P = 0.036), hyperlipidemia (53.9% vs. 34.2%, P = 0.005), and smoking (60.7% vs. 32.4%, P < 0.001) were also more prevalent among patients with severe lesions. Multivariate analysis identified male gender (odds ratio (OR): 4.167, 95% confidence interval (CI): 1.798–9.655, P = 0.001), hyperlipidemia (OR: 2.342, 95% CI: 1.106–4.959, P = 0.026), and the presence of fQRS (OR: 5.814, 95% CI: 2.478–13.645, P < 0.001) as independent predictors of severe stenosis. ROC analysis demonstrated moderate diagnostic performance for fQRS (area under the curve (AUC): 0.745, 95% CI: 0.673–0.816, P < 0.001), yielding a sensitivity of 65.2% and a specificity of 83.8%.

**Conclusion:**

The presence of fQRS is strongly and independently associated with anatomically severe stenosis in patients with intermediate lesions on CCTA. Incorporating baseline fQRS evaluation may help refine clinical decision-making and narrow the diagnostic “gray zone.”

## Introduction

Cardiovascular diseases, particularly coronary artery disease (CAD), remain the leading causes of morbidity and mortality worldwide [1]. Consequently, accurate diagnosis and risk stratification are crucial for optimal treatment planning. Coronary computed tomography angiography (CCTA) is widely recommended for the anatomical evaluation of stable chest pain in patients with low-to-intermediate risk [2], leveraging its high negative predictive value to reduce unnecessary invasive coronary angiography (ICA) [3].

However, the correlation between CCTA and ICA remains imperfect for intermediate-grade stenoses (40–70%). Severe calcified plaques frequently cause overestimation of stenosis severity, whereas non-calcified lipid-rich lesions may be underestimated [4, 5]. This diagnostic discrepancy can result in either redundant invasive interventions or missed significant lesions [6, 7]. While functional modalities such as fractional flow reserve derived from CT (FFR-CT) or single-photon emission computed tomography (SPECT) can clarify this gray zone, they impose substantial practical and economic burdens, underscoring the need for simpler, universally accessible markers.

Fragmented QRS (fQRS) on a standard 12-lead electrocardiography (ECG)—defined as additional notching within the QRS complex—reflects heterogeneous ventricular activation caused by myocardial scarring, fibrosis, or regional ischemia [8, 9]. Beyond its established association with reduced ventricular function, arrhythmic events, and increased mortality [9–12], fQRS correlates with hemodynamically significant ischemia and overall CAD complexity [10, 11].

Therefore, this study investigated whether resting ECG fQRS predicts anatomically severe stenosis (≥ 70%) on ICA in patients presenting with CCTA-detected intermediate lesions. We hypothesized that integrating this easily accessible electrocardiographic parameter could improve patient selection for invasive evaluation.

## Materials and Methods

### Study design and patient selection

This retrospective cross-sectional study screened patients with intermediate coronary stenosis (40–70%) identified on CCTA between January 2022 and June 2025. A total of 200 patients who underwent subsequent ICA within 3 months were enrolled. Inclusion criteria comprised an age ≥ 18 years, CCTA-confirmed intermediate stenosis, completed ICA, and an interpretable baseline 12-lead ECG. Exclusion criteria were prior revascularization, acute coronary syndrome, complete bundle branch block, pacemaker rhythm, second- or third-degree atrioventricular block, active infection, or poor-quality electrocardiographic records. Patients with left main or proximal left anterior descending artery stenosis ≥ 50% were also excluded.

### Ethical statements

This retrospective study was conducted in accordance with the Declaration of Helsinki and approved by the Ethics Committee of Harran University Faculty of Medicine (Approval Date: September 15, 2025; Approval Number: HRÜ/25.15.36).

### ECG and fQRS criteria

Standard 12-lead ECGs (10 mm/mV, 25 mm/s) were obtained a median of 7 days (interquartile range (IQR): 3–14) prior to ICA, with no interval clinical events reported. Two cardiologists blinded to clinical data independently evaluated the tracings (interobserver reliability, κ = 0.82), resolving disagreements (8%) via consensus. In accordance with established criteria, fQRS was defined as the presence of various RSR' patterns or distinct S-wave notching in at least two contiguous leads corresponding to a specific coronary territory. Patients with an intraventricular conduction delay or incomplete bundle branch block were excluded from the analysis.

### CCTA

All CCTA examinations were performed using a 320-detector CT scanner (Canon Medical Systems, Otawara, Japan). Patients received sublingual nitroglycerin and, when baseline heart rate exceeded 70 bpm, oral metoprolol. Contrast enhancement was achieved by injecting 60 mL of iodixanol (320 mg/mL) at a rate of 5 mL/s, followed by a saline flush. Curved planar and maximum intensity projection reconstructions were independently evaluated by two radiologists, with discrepancies arbitrated by a third senior reviewer.

### ICA

ICA was performed within 3 months of CCTA via the standard Judkins technique. Stenosis severity was visually quantified by two independent interventional cardiologists who were blinded to the clinical and electrocardiographic findings. Patients were categorized into severe stenosis (≥ 70%) and non-severe stenosis (< 70%) cohorts based on the maximum luminal narrowing observed in the target vessel.

### Statistical analysis

Statistical analyses were executed using SPSS version 28.0. Continuous variables were compared using Student’s *t*-test or the Mann–Whitney U test, as appropriate, while categorical variables were assessed via the Chi-square test. Univariate and multivariate logistic regression analyses identified independent predictors of severe stenosis. Receiver operating characteristic (ROC) curves evaluated the diagnostic performance of fQRS. Statistical significance was defined as a P-value < 0.05.

## Results

### Study population and baseline characteristics

The final cohort consisted of 200 patients with CCTA-defined intermediate stenosis (mean age: 58.4 ± 10.2 years; 54.5% male) who underwent invasive evaluation. Invasive angiography confirmed severe stenosis (≥ 70%) in 89 patients (44.5%) and non-severe stenosis (< 70%) in 111 patients (55.5%). Baseline fQRS was identified in 76 patients (38.0%) across the entire study population.

### Comparison of demographic and clinical characteristics

Baseline demographic, clinical, and laboratory parameters are summarized in Table 1. Group 1 (≥ 70% stenosis) exhibited a significantly higher proportion of male patients and a higher body mass index (BMI) than group 2 (< 70% stenosis) (67.4% vs. 36.9%, P < 0.001; 29.9 ± 2.4 vs. 28.7 ± 3.2 kg/m^2^, P = 0.002, respectively). Notably, the presence of fQRS was significantly more frequent in group 1 than in group 2 (65.2% vs. 16.2%, P < 0.001). Regarding traditional cardiovascular risk factors, diabetes mellitus (36.0% vs. 22.5%, P = 0.036), hyperlipidemia (53.9% vs. 34.2%, P = 0.005), and active smoking (60.7% vs. 32.4%, P < 0.001) were significantly more prevalent in the severe stenosis cohort. No significant differences were observed regarding hypertension, chronic obstructive pulmonary disease, or family history of CAD (P > 0.05).

**Table 1:** Baseline Demographic, Clinical, and Laboratory Characteristics of the Study Population

Variables	Group 2 (< 70% stenosis, n = 111)	Group 1 (≥ 70% stenosis, n = 89)	P
Age (years)	58.6 ± 7.9	56.8 ± 11.3	0.392
Gender, male (n, %)	41 (36.9)	60 (67.4)	< 0.001*
Weight (kg)	80.1 ± 6.9	86.5 ± 6.2	< 0.001*
Height (cm)	167.5 ± 6.5	170.2 ± 5.9	0.006*
BMI (kg/m^2^)	28.7 ± 3.2	29.9 ± 2.4	0.002*
Fragmented QRS presence (n, %)	18 (16.2)	58 (65.2)	< 0.001*
Hypertension (n, %)	67 (60.4)	56 (62.9)	0.711
Diabetes mellitus (n, %)	25 (22.5)	32 (36.0)	0.036*
COPD (n, %)	18 (16.2)	13 (14.6)	0.755
Family history (n, %)	56 (50.5)	43 (48.3)	0.764
Coronary CT bridge presence (n, %)	22 (19.8)	14 (15.7)	0.454
Hyperlipidemia (n, %)	38 (34.2)	48 (53.9)	0.005*
Smoking (n, %)	36 (32.4)	54 (60.7)	< 0.001*
Glucose (mg/dL)	130.5 ± 57.7	139.3 ± 63.4	0.111
Urea (mg/dL)	28.1 ± 11.2	27.3 ± 11.0	0.978
Creatinine (mg/dL)	0.82 ± 0.19	0.82 ± 0.19	0.840
HbA1c (%)	6.62 ± 1.48	6.69 ± 1.58	0.861
TSH (mIU/L)	2.54 ± 1.47	2.55 ± 1.52	0.879
T4 (ng/dL)	14.4 ± 2.3	14.6 ± 2.5	0.436
Triglyceride (mg/dL)	187.4 ± 122.0	153.8 ± 67.0	0.088
LDL (mg/dL)	112.6 ± 27.4	110.0 ± 22.3	0.700
Hemoglobin (g/dL)	15.5 ± 2.2	15.1 ± 2.1	0.504
Platelet (× 10^3^/µL)	287.4 ± 73.7	266.1 ± 72.5	0.043*
Acetylsalicylic acid (n, %)	32 (29)	25 (28)	0.206
Beta blocker (n, %)	24 (22)	23 (25)	0.394
Statins (n, %)	29 (26)	24 (27)	0.276

Data are presented as mean ± standard deviation or n (%). *P-values indicate statistical significance (P < 0.05). BMI: body mass index; COPD: chronic obstructive pulmonary disease; CT: computed tomography; HbA1c: glycated hemoglobin; LDL: low-density lipoprotein; T4: thyroxine; TSH: thyroid-stimulating hormone.

### Laboratory parameters

Routine laboratory markers—including hemoglobin, leukocyte counts, creatinine, fasting blood glucose, and lipid profiles—were comparable between the two groups (P > 0.05). However, baseline platelet counts were significantly lower in patients with severe stenosis than in those with non-severe lesions (266.1 ± 72.5 × 10^3^/µL vs. 287.4 ± 73.7 × 10^3^/µL, P = 0.043; Table 1).

### Univariate and multivariate logistic regression analysis

Univariate and multivariate binary logistic regression models were constructed using clinically relevant variables and established cardiovascular risk factors (Table 2). In the fully adjusted multivariate model, male gender (odds ratio (OR): 4.167, 95% confidence interval (CI): 1.798–9.655, P = 0.001), hyperlipidemia (OR: 2.342, 95% CI: 1.106–4.959, P = 0.026), and the presence of fQRS (OR: 5.814, 95% CI: 2.478–13.645, P < 0.001) emerged as robust independent predictors of anatomically severe coronary stenosis (≥ 70%).

**Table 2:** Univariate and Multivariate Logistic Regression Analysis for Predictors of Severe Stenosis (≥ 70%)

Variable	Univariate model			Multivariate model		
	OR	95% CI	P	OR	95% CI	P
Male gender	3.512	1.980–6.230	< 0.001*	4.167	1.798–9.655	0.001*
BMI (kg/m^2^)	1.071	0.977–1.175	0.147	0.918	0.811–1.039	0.177
Hypertension	1.304	0.773–2.199	0.320	1.151	0.586–2.258	0.684
Diabetes mellitus	1.928	1.041–3.570	0.036	1.087	0.471–2.506	0.846
Smoking	3.047	1.789–5.191	< 0.001*	1.429	0.614–3.323	0.408
Hyperlipidemia	2.539	1.481–4.352	0.001*	2.342	1.106–4.959	0.026*
Family history	1.296	0.776–2.165	0.321	0.672	0.360–1.256	0.214
Fragmented QRS presence	4.998	2.677–9.331	< 0.001*	5.814	2.478–13.645	< 0.001*
Triglyceride	0.997	0.994–1.001	0.305	0.998	0.995–1.001	0.221
LDL	1.006	0.996–1.016	0.267	0.990	0.977–1.003	0.149

*P-values indicate statistical significance (P < 0.05). BMI: body mass index; LDL: low-density lipoprotein; OR: odds ratio; 95% CI: confidence interval.

### ROC curve analysis and diagnostic performance

**Figure 1:**
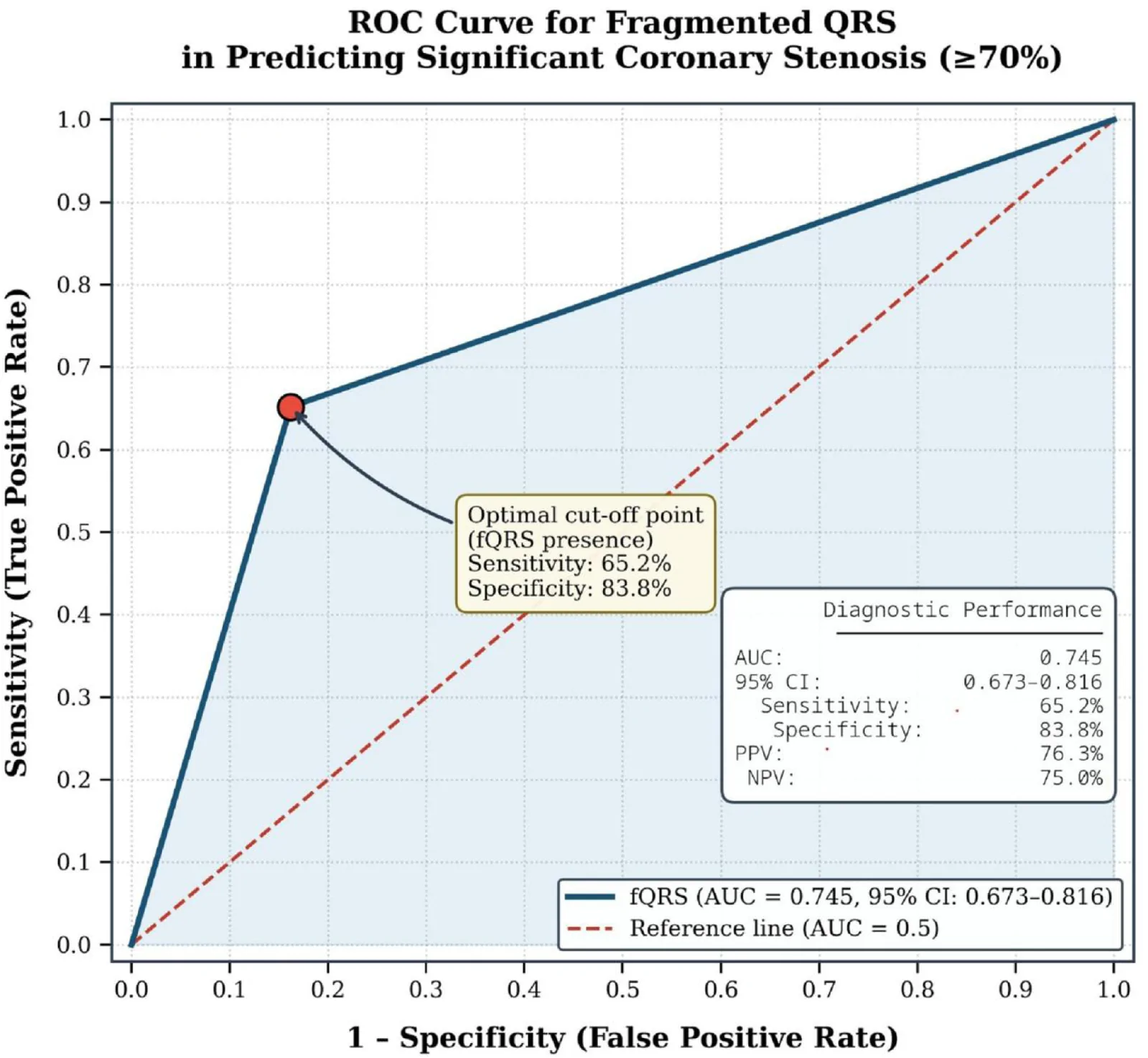
Receiver operating characteristic (ROC) curve analysis of fragmented QRS (fQRS) for predicting anatomically significant coronary stenosis (≥ 70%) on invasive coronary angiography.

ROC curve analysis demonstrated that the presence of fQRS significantly predicted severe angiographic stenosis with moderate diagnostic performance (Fig. 1). The area under the curve (AUC) was 0.745 (95% CI: 0.673–0.816, P < 0.001). At the optimal cut-off point, determined by maximizing the Youden index, fQRS demonstrated a sensitivity of 65.2%, a specificity of 83.8%, a positive predictive value of 76.3%, and a negative predictive value of 75.0% for identifying severe narrowing in the target vessel (Table 3).

**Table 3:** Diagnostic Performance of fQRS for Predicting Severe Stenosis (≥ 70%) in ROC Curve Analysis

	< 70% Stenosis (n = 111)	≥ 70% Stenosis (n = 89)	Total
fQRS (−)	93	31	124
fQRS (+)	18	58	76
Total	111	89	200

Measure	Value
AUC (95% CI)	0.745 (0.673–0.816)
P-value	< 0.001
Sensitivity	65.2%
Specificity	83.8%
Positive predictive value	76.3%
Negative predictive value	75.0%

## Discussion

This study demonstrates that a resting ECG fQRS is strongly and independently associated with anatomically significant coronary stenosis (≥ 70%) on ICA in symptomatic patients previously classified as having intermediate lesions via CCTA. Along with male gender and hyperlipidemia, the presence of baseline fQRS served as an independent predictor of severe disease, showcasing moderate diagnostic performance with high specificity (83.8%) and a favorable positive predictive value (76.3%).

Mechanistically, fQRS reflects distorted, heterogeneous ventricular depolarization induced by myocardial scarring, localized fibrosis, or chronic ischemia [8, 9]. Ischemic pathways alter regional conduction, producing fragmentation via functional delays rather than permanent cell death—a phenomenon often explained by myocardial stunning or hibernation [8–10]. Beyond myocardial scarring, fQRS may also arise from functional conduction delays due to acute or chronic ischemia, potentially reflecting hibernating or stunned myocardium. Importantly, the progression from stable ischemia to severe anatomical stenosis is not merely a local vascular phenomenon but is influenced by systemic metabolic and proteostatic disturbances. Recent evidence suggests that aging-related deterioration in respiratory and metabolic homeostasis, along with chronic subclinical acid-base imbalances, may contribute to endothelial dysfunction and vascular remodeling [13, 14]. These systemic processes create a permissive environment for the development of severe coronary lesions, and fQRS might serve as an electrical integrator of these multifaceted pathological changes.

While fQRS is widely recognized for predicting structural scars and adverse long-term prognoses [12], the high OR observed in our multivariate model suggests that fragmentation also serves as an electrical surrogate for a substantial myocardial substrate at risk distal to an anatomically severe lesion.

Our findings align with and expand upon prior literature. Korkmaz et al reported that fQRS independently predicts FFR-defined functional ischemia in moderate coronary lesions [11]. Our data extend this paradigm by confirming a direct correlation with anatomical severity on conventional angiography. Interestingly, while Viriyanukulvong et al observed low sensitivity but high specificity for fQRS in detecting cardiac magnetic resonance-confirmed myocardial scars [12], our study yielded a higher sensitivity of 65.2%. This discrepancy supports the hypothesis that acute or chronic ischemic mechanisms, distinct from established dense scars, contribute heavily to QRS fragmentation during active coronary insufficiency. Furthermore, Tabatabaei et al noted that combining fQRS with advanced strain imaging enhances the non-invasive detection of significant stenosis [15], validating the clinical utility of integrating basic electrical markers with anatomical imaging.

Regarding diabetes mellitus, which was more prevalent in the severe stenosis group, our findings align with the known heightened cardiovascular risk in diabetic patients. Beyond its well-established micro- and macrovascular complications, recent molecular studies have highlighted the potential role of metal-homeostasis disturbances and protein aggregation in diabetic vascular pathology [16, 17]. While our study did not investigate these molecular pathways, these observations provide a compelling biological rationale for the increased risk of severe coronary disease observed in our diabetic cohort.

The clinical utility of CCTA is often bottlenecked by its intermediate diagnostic range. Extensive calcification artifacts and complex plaque morphology regularly cause luminal overestimation or underestimation [4, 5]. This limitation was highlighted by Kim et al, who reported a modest 18% agreement rate between initial CCTA assessments and subsequent ICA findings in moderate stenoses [6]. Similarly, insights from the ISCHEMIA trial demonstrated that CCTA resulted in a 25% overestimation and a 20% underestimation of significant disease [18]. In our cohort, 44.5% of CCTA-intermediate lesions represented true angiographic stenosis ≥ 70%, highlighting the clinical imperative for accessible risk-stratification tools.

As a simple, cost-effective, and universally available parameter, fQRS represents an attractive adjunctive marker in everyday practice. While it cannot replace advanced functional modalities like FFR-CT, it may serve as an effective clinical gatekeeper to prioritize symptomatic patients for invasive evaluation.

Several clinical observations in our trial warrant mention. The lack of association between a family history of CAD and severe stenosis suggests that genetic predisposition primarily drives overall CAD development rather than specific lesion severity. Additionally, low-density lipoprotein (LDL) cholesterol levels did not differ significantly between groups despite a higher prevalence of hyperlipidemia in the severe stenosis cohort. This paradox is likely explained by the identical rates of baseline statin therapy between the two groups (27% vs. 26%, P = 0.276). Future investigations should evaluate non-high-density lipoprotein (HDL) and lipoprotein(a) to better delineate these lipid profiles.

Our findings add to a growing body of literature evaluating inexpensive, readily available electrocardiographic markers for improving patient stratification before ICA. While parameters such as T-wave alternans or QTc dispersion have been explored, fQRS offers the advantage of being easily and rapidly identifiable on a standard 12-lead ECG without the need for specialized software or signal-averaging techniques. Further comparative studies are warranted to determine the most effective combination of these markers for clinical use.

### Limitations

This study has several limitations. First, its retrospective, single-center design precludes establishing definitive causality and limits global generalizability. Second, verification bias is inherent, as we only enrolled patients clinically referred for ICA based on symptom burden or positive stress tests, thereby selecting a higher-risk cohort. Consequently, our diagnostic metrics reflect clinical utility in symptomatic individuals rather than a general screening population. Third, the absence of functional lesion assessments (e.g., FFR, iFR) prevents a direct correlation with true ischemia, as anatomical narrowing does not always mirror hemodynamic significance. Fourth, although baseline ECGs were recorded a median of 7 days prior to ICA, fQRS represents a marker of chronic electrical remodeling and is unlikely to fluctuate acutely. Fifth, the diagnostic performance of fQRS in our study, while statistically significant, demonstrated only moderate sensitivity (65.2%). This relatively low sensitivity, coupled with the fact that fQRS can also be observed in other conditions such as cardiomyopathies or prior silent myocardial fibrosis unrelated to the target lesion, suggests that this parameter should be used as an adjunctive tool rather than a sole diagnostic test. Its specificity, therefore, might be lower in an unselected, broader patient population. Sixth, while interobserver agreement for fQRS assessment was high in our core-lab setting (κ = 0.82), variability may be greater in routine clinical practice, potentially affecting its generalizability. Seventh, ejection fraction (EF), a potential confounder and a marker of long-standing ischemia or prior infarction, could not be reliably assessed in this retrospective cohort. Future prospective studies should include EF to evaluate its interaction with fQRS and lesion severity. Finally, the optimal ROC cut-off point for fQRS was determined using the Youden index to maximize sensitivity and specificity, but this threshold requires external validation in independent cohorts.

### Conclusion

A standard 12-lead ECG fQRS is an independent predictor of anatomically significant stenosis (≥ 70%) on ICA in patients presenting with CCTA-detected intermediate lesions. Given its high specificity and accessibility, this simple parameter can help narrow the diagnostic “gray zone,” optimizing patient triage for invasive angiography. These findings warrant validation in large-scale prospective trials to fully integrate fQRS into clinical decision-making algorithms.

AUC: area under curve; 95% CI: confidence interval; ROC: receiver operating characteristics.

## Data Availability

The datasets used and/or analyzed during the current study are available from the corresponding author upon reasonable request.

## Abbreviations

AUC: area under the curve
BMI: body mass ındex
CAD: coronary artery disease
CCTA: coronary computed tomography angiography
CI: confidence ınterval
ECG: electrocardiogram
fQRS: fragmented QRS
ICA: invasive coronary angiography
OR: odds ratio
ROC: receiver operating characteristic
